# Revealing Underdrawings in Wall Paintings of Complex Stratigraphy with a Novel Reflectance Photoacoustic Imaging Prototype

**DOI:** 10.3390/jimaging7120250

**Published:** 2021-11-24

**Authors:** Antonina Chaban, George J. Tserevelakis, Evgenia Klironomou, Raffaella Fontana, Giannis Zacharakis, Jana Striova

**Affiliations:** 1 National Institute of Optics INO-CNR, 50125 Florence, Italy; raffaella.fontana@ino.cnr.it (R.F.); jana.striova@cnr.it (J.S.); 2 Institute of Electronic Structure and Laser, Foundation for Research and Technology Hellas, 70013 Heraklion, Crete, Greece; tserevel@iesl.forth.gr (G.J.T.); eklironomou@physics.uoc.gr (E.K.); zahari@iesl.forth.gr (G.Z.)

**Keywords:** photoacoustic, infrared reflectance, non-invasive, underdrawings, wall paintings, complex stratigraphy

## Abstract

Revealing precious hidden features by a completely non-invasive approach is one of the crucial issues in the Heritage Science field. In this regard, concealed fresco paintings still represent an analytical challenge. This paper addresses the specific issue in wall painting diagnostics by the photoacoustic (PA) imaging technique, already proven to be efficient in revealing underdrawings and internal stratigraphy in movable paintings on paper and canvas. A newly set-up reflection PA prototype was applied here for the first time to probe the charcoal, graphite and sinopia hidden sketch drawings in concealed (gypsum, limewash, overpainted) wall paintings. The results presented here push forward the frontiers of the PA imaging technique and point to its potential effectiveness of revealing hidden underdrawings in historical wall paintings with complex stratigraphy.

## 1. Introduction

Numerous historical wall paintings remain in part or completely covered by subsequent layers, including retouchings, overpaints, mortar coats, etc. A rediscovery of the hidden features of wall paintings is a frequent and challenging case in conservation. A reliable non-invasive diagnostic procedure, as the first step, is crucial to a painting’s correct characterization and eventual safe uncovering. There is a need for a robust methodology that is capable of addressing all the relevant art historical, material and conservation queries prior to any intervention on such multilayered wall paintings.

Valuable hidden features may include original underdrawing or even an underpainting, realized in fresco, *mezzo* fresco or secco techniques. The *buon* fresco (or true fresco) is realized by applying the sketch and paint (pigment mixed with water without a binding agent) on lime mortar while it is still wet. *A secco* method foresees the pigment application on a dry surface and therefore requires a binding medium (e.g., egg tempera, oil, glue, acrylic binder) to attach the pigment to the wall. *A mezzo fresco* indicates a wall painting technique where the pigment is applied with water onto the humid (almost dry) mortar, so that it only slightly penetrates into the wall. Detailed information about wall painting execution techniques can be found elsewhere [1,2,3,4,5]. Further interventions could see the use of limewash coat to conceal the original sketch or the changes of the final appearance of a painting by retouching (further paint layer applied *a secco*). Heritage experts face even such cases where original paintings were covered by subsequent mortar and paint layers due to changes in taste, religious belief, ownership or alterations of the building. Indeed, many examples of precious paintings or sketch drawings covered during history are reported [6,7,8,9,10,11].

As a matter of fact, many historical paintings are characterized by often unknown internal stratigraphy. Near-infrared (NIR) reflectance imaging has become the most consolidated method in the detection of underdrawings underneath the depicted surface [12,13,14,15,16,17,18,19]. This method exploits an increased penetrability of near-infrared radiation through a pictorial layer (most of artistic pigments have low scattering/absorption in the near-infrared range) and strong optical absorption of sketch materials (charcoal, iron-gall ink etc.), in the condition of a strong diffuse near-infrared reflectance from the ground. Many studies can be found in the literature on NIR imaging used to reveal underdrawings in fresco and secco wall paintings underneath retouched areas, while little can be found on deeper concealed features (covered by limewash, concealing mortar layers etc.). A dedicated study has already proven the efficiency of NIR imaging to reveal iron-gall ink and carbon-based sketches on mock-ups with a limewash layer up to 200 μm thick, by selecting the most appropriate wavelength [20].

The literature reports studies on infrared thermography that revealed hidden wall decoration layers, including wall frescoes [10]. Further rapidly developing techniques applied to revealing hidden features in multilayered paint stratigraphy include THz Imaging [21,22,23,24], Optical Coherence Tomography [25,26,27] and the newly introduced epi-illumination Photoacoustic (PA) Imaging [28]. In terms of spatial resolution and image contrast, the latter showed a particularly strong potential for the matter of this study. In previous years, PA imaging was applied to cultural heritage objects only in transmission mode [28,29,30,31,32,33]. Such configuration cannot be used on wall paintings, since the pictorial layer on its backside is bonded to the structural support (wall, vault etc.). The novel epi-illumination PA imaging set up proved its efficiency in detecting underdrawings in simulated multilayered artworks [28], pointing to its applicability in a wide range of Cultural Heritage objects of arbitrary forms and shapes. Here, we tested it on specifically designed wall painting mock-ups, prepared following the historical methodologies. In particular, the scope of this work was to evaluate the effectiveness of the new epi-illumination PA imaging set-up to detect the sketch drawings in simulated wall paintings. These features are hidden under a secco paint layer or under both the fresco paint and the concealing limewash or gypsum coats of two different thicknesses.

We compared and complemented here the PA imaging results with the information obtained by research-grade near-infrared reflectance (NIR) imaging, operated in 380–2500 nm. The information on the layer thickness was achieved by laser scanning profilometry.

## 2. Experimental

### 2.1. Methods

#### 2.1.1. Photoacoustic Imaging Set-Up

The reflection-mode PA imaging apparatus employs a Q-switched Nd:YAG laser emitting infrared radiation at 1064 nm for the excitation of a PA signal. The object is irradiated from its front side to generate a laser-induced ultrasound from the underlying hidden sketch regions. The PA waves are transmitted through the overlying layers and water prior to their detection in reflectance configuration by a broadband spherically focused piezoelectric transducer. The integration of immersion ultrasonic detection (e.g., here in a water medium) minimizes acoustic reflection losses at poorly matched interfaces such as between paint layers and air [34,35]. The signals are subsequently enhanced by two low-noise radio frequencies (RF) amplifiers prior digitization and recording of PA waveforms by an oscilloscope. The image is formed through raster scanning along the analyzed surface using a set of high-precision XY motorized stages. The recorded waveforms were averaged two times for signal-to-noise ratio (SNR) improvement, transferred to a computer, and band-passed between 100 kHz and 30 MHz for high-frequency noise elimination before the estimation of the peak-to-peak PA amplitude value, providing the contrast of the resulting 8-bit images. The pulse energy at the object’s plane surface was kept below 2.3 mJ (corresponding surface fluence for spot size 1 mm^2^ is less than 0.3 J/cm^2^), following the results of a preliminary investigation of the optimum irradiation parameters (pulse energy, number of averaging waveforms), required to provide sufficient SNR levels without the presence of any apparent photodamage effects. Each mock-up was placed at the bottom of a 3D-printed sample holder filled with distilled water, serving as an immersion medium for the efficient propagation and subsequent detection of PA signals. The scanned regions had dimensions ranging between 1.5 × 1.5 to 2.5 × 2.5 cm^2^ and were sampled using a pixel size of 300 × 300 μm^2^. The total time required for the recording of a PA image ranged from 40 min to 2 h. Control and synchronization of the PA imaging system was carried out by means of a custom-developed software, while image-processing operations were performed in ImageJ and MATLAB programming environment. The pixel size of all PA images presented in this study is 300 × 300 μm^2^. Further technical details on the PA imaging set-up can be found in [28].

#### 2.1.2. Visible-Near-Infared (VIS-NIR) Reflectance Imaging

The multispectral VIS-NIR scanner developed at the National Institute of Optics acquires the backscattered radiation from the measured surface. It scans with a spatial sampling of 4 point/mm (250 μm), generating 32 monochromatic images in the visible (from 380 to 780 nm) and in the infrared region (790 to 2500 nm) with a spectral sampling step of 20–30 and 50–100 nm, respectively [36,37]. The VIS-NIR multispectral scanner illuminates the surface and collects the backscattered radiation in a point-by-point modality. The optical head bears the lighting system and the collecting. The instrument allows to scan continuously an area of up to 1 m^2^ within 3 h, exploiting two orthogonally mounted high-precision linear-translation stages, equipped with optical encoders. The acquisition time for a single point is about 1 msec. The constant motion of the optical head prevents the surface of the painting from being heated significantly. It works at a distance of 12 cm from the object’s surface in a 45°/0° (illumination/detection) configuration according to CIE standards. The system is operated through a custom-developed software, with a simultaneous control of movement of scanner head, autofocus and images acquisition. The pixel size of all NIR images presented in this study is 250 × 250 μm^2^.

Processing in ImageJ was applied first to enhance the PA result. Subsequently, the same image processing function was applied to NIR images for comparison of the experimental results.

#### 2.1.3. Conoscopic Microprofilometry

A conoscopic microprofilometer acquires a collection of vertices over a regular grid, creating a 3D model of the measured surface. The working distance from the surface is 4 cm, with a dynamic of 8 mm (depth of field) whereas the acquisition frequency is about 400 points/s. The maximum scanning area is 30 × 30 cm with 1 μm axial and 20 μm lateral resolution [38,39]. It is operated through a custom-developed software. Laser scanning conoscopic microprofilometry provided a topographic map of the mock-up’s surface, supplying height (z) values, which were used here for thickness and surface topography measurements. The time required for the recording of an area 12 × 12 cm was of 1 h 45 min. The thickness of hiding layers was calculated through subtraction of 3D topography maps acquired on the mock-ups before and after application of painting layers and coats.

### 2.2. Experimental Mock-Ups

**Figure 1:**
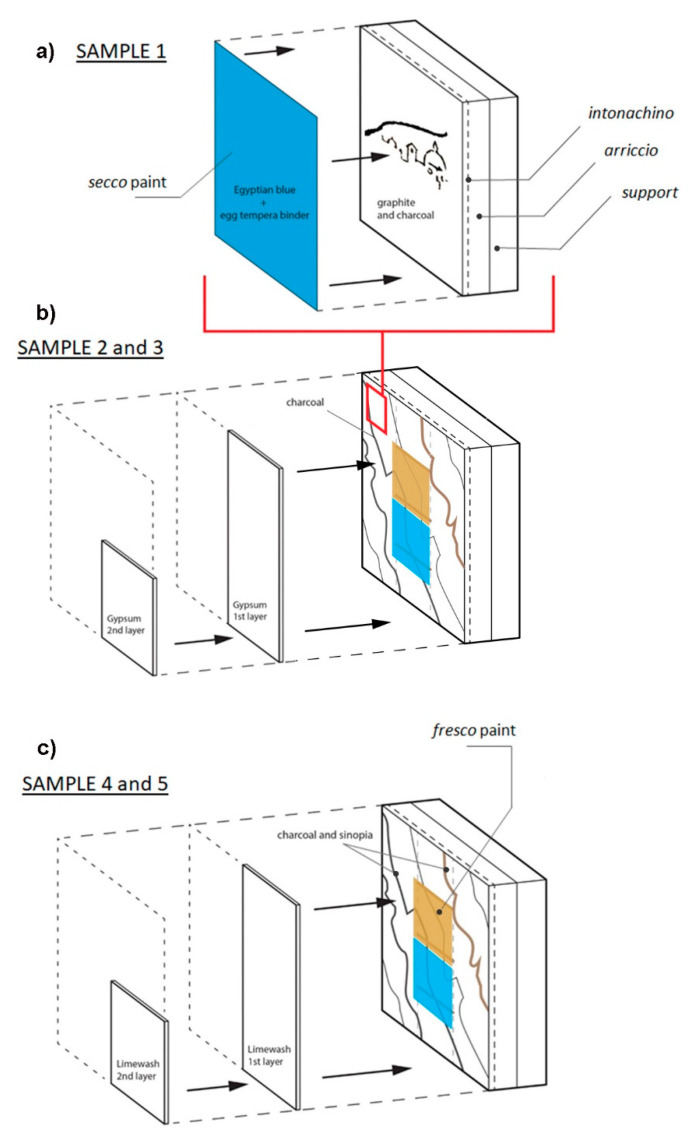
Stratigraphy scheme of the wall painting mock-ups: (**a**) Sample 1, containing underdrawings hidden under Egyptian blue secco paint layer; (**b**) Sample 2, which contains underdrawings, covered by 1 and 2 gypsum coats, and Sample 3, which contains underdrawings, covered by fresco and by 1 gypsum coat. (**c**) Sample 4, which contains underdrawings, covered by 1 and 2 limewash coats, and Sample 5, which contains underdrawings, covered by fresco and 1 limewash coat.

The effectiveness of the PA imaging technique was evaluated on an ad-hoc prepared mock-ups simulating a real wall painting. In particular, the analyzed hidden features and top layers are described in Table 1. The mock-ups stratigraphy is illustrated in Figure 1.

The mock-ups were prepared according to the traditional recipe of true fresco technique [1,2], within the premises of Accademia dell’Affresco in Padua (Italy). After 1 year of natural carbonatation, all the hiding layers (secco paint, gypsum and limewash) were applied onto the mock-ups’ surfaces at the Opificio delle Pietre Dure in Florence (Italy).

*Fresco mock-ups*: a thick layer (1 cm) of medium coarse mortar called *arriccio* (a mix of slaked lime and medium grain sand (1:2) with water) was applied on a lightweight wood-fiber support. Subsequently, a thin layer (2–3 mm) of fine coarse mortar called *intonachino* (a mix of slaked lime and fine grain sand (1:2) with water) was applied upon it.

*Outline/sketch drawings:* In order to simulate the characteristic fresco underdrawings, a black charcoal pigment *nero carbone* and sinopia (both Dolci, Verona) were applied on the lime mortar while it was still wet. The sketch was transferred to the mortar surface using the traditional for fresco paintings *spolvero* (pouncing) technique. Additional graphite sketches (Koh-I-Noor Hardtmuth pencil, hardness level 2B) were realized on the dry mortar surface.

*Pigments*: Two characteristic for fresco (stable in alkaline environment) pigments—Egyptian blue (EB) and raw sienna (RS) [40]—were applied on a fresh mortar, shortly after sketch.

*Hiding coats:* EB was applied *a secco* using egg yolk tempera binder (egg yolk and distilled water, 1:1) on the dry mortar surface to simulate simple retouching or overpaint (Sample 1). Gypsum (calcium sulphate dihydrate CaSO_4_. 2H_2_O, bound with rabbit glue dissolved in water in proportion 1 g:12 mL) and limewash (lime, water with a small addition of milk) coats were applied on dry substrate to conceal the underdrawings, respectively, in Samples 2 and 3 and in Samples 4 and 5. Gypsum and limewash coats were applied by brush in one and two layers to obtain two different thicknesses as specified in Table 1.

## 3. Results

### 3.1. Imaging Charcoal and Graphite Outlines under Tempera Paint: Sample 1

**Figure 2:**
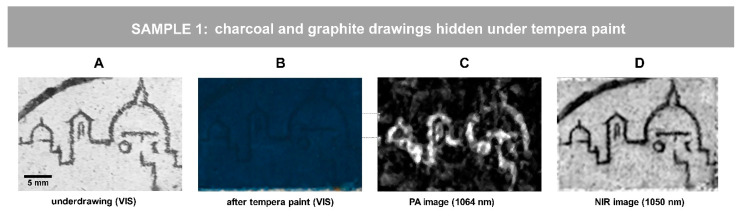
The charcoal (line in upper left corner) and graphite (city skyline) traits on the mortar: visible images (**A**) prior to paint application and (**B**) after Egyptian blue tempera application (secco overpaint); (**C**) a PA image and (**D**) NIR reflectance image of (**B**). The scale bar in (**A**) applies to all the images.

A graphite drawing representing a city skyline as well as the charcoal line (Figure 2A) were hidden under a 70-micron thick Egyptian blue paint layer, applied with egg tempera binder (Figure 2B). The raw PA and NIR images were processed in equal way by the means of ImageJ, applying 1.0% contrast stretching. The PA image (Figure 2C) of the painted sample, obtained in reflection mode, clearly reveals the sketch drawing made by graphite pencil directly on the dried mortar. Both graphite and charcoal traits are revealed in the NIR reflectogram (Figure 2D). However, graphite has a significantly higher absorption than charcoal at 1064 nm [29,41,42]. Since the image contrast in PA image is relative per scanned area, the charcoal line presents in this specific case a too low contrast to be visible in the PA image. For revealing low contrast details in a PA image, application of post-processing algorithms or additional area scans might be needed. These tests lie beyond the aims of the first experimentation and are expected to be addressed by future studies.

### 3.2. Imaging Charcoal and Sinopia Drawings under Gypsum Coat: Samples 2 and 3

**Figure 3:**
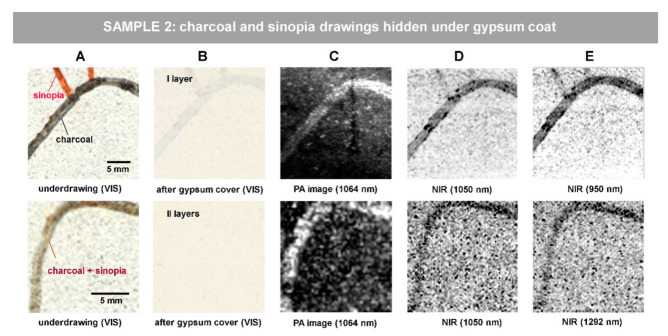
Revealing hidden sketch drawings under gypsum coat in Sample 2: (**A**) visible image prior to gypsum application; (**B**) visible image after gypsum coat application; (**C**) PA imaging result; (**D**,**E**) NIR reflectance images. The scale bar in (**A**) is valid for all the images of the row.

Visible images of Sample 2 are shown in Figure 3 along with the relevant representative results. In top raw, sinopia and charcoal traits on substrate (Figure 3A) are hidden by a 80-micron thick gypsum layer (Figure 3B); in bottom raw, mixture of sinopia and charcoal is covered by 190-micron thick gypsum layers (Figure 3B). Both PA and NIR images were processed in an equal way by the means of ImageJ. The function of 1.0% contrast stretching and 0.6 gamma correction were applied to the PA and NIR images in the top raw and 1.0% contrast stretching with a min value of 10.826 to the PA and NIR images in the bottom raw. The min value corresponds to the lower limit of the display range for the processed 16-bit images (brightness range: 0–65.536), so that all pixels with a brightness equal to or below 10.826 were set to zero (total black). The representative images of the PA and NIR investigations are displayed respectively in Figure 3C and Figure 3D,E. For the NIR investigation, first, the reflectogram centered at 1050 nm (1000–1100 nm) is shown in Figure 3D due to the overlap with the excitation wavelength exploited by PA. Then, the last column shows the highest contrast NIR reflectogram, at 950 nm and at 1292 nm, for the two areas, respectively. As for the results shown in the top raw, the charcoal line is clearly distinguished by both PA and NIR methods, while sinopia only by NIR (Figure 3C–E).

The PA technique demonstrates a good capability in revealing charcoal traits hidden under the gypsum coats (Figure 3C). In the top raw (Figure 3), a 80-micron thick gypsum layer covers both charcoal and sinopia lines. A charcoal line hidden under one and two layers of gypsum cover (respectively 80 and 190 μm thick) is detectable by the PA technique, as shown in Figure 3C. This method presents a good capability in revealing charcoal under the gypsum coats and even allows clear detectability of charcoal in lower concentration (mixed with sinopia) under two layers of gypsum (thickness 190 μm). At the same time, sinopia is not detectable by photoacoustic imaging at the exploited wavelength (1064 nm) but is detectable by near-infrared reflectance imaging. The combined PA imaging and NIR reflectance imaging approach is shown to be helpful here in the detection of both underdrawing materials (charcoal and sinopia) and making a hypothesis about its type.

**Figure 4:**
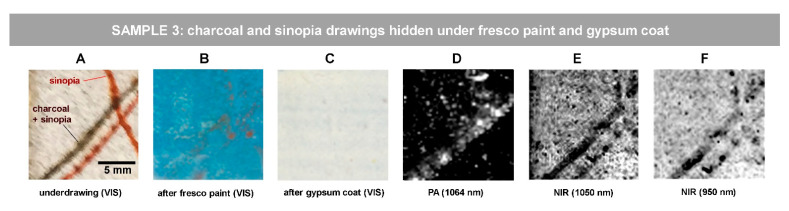
Revealing hidden sketch drawings under fresco paint and gypsum coat in Sample 3: visible images of (**A**) initial drawing on the substrate; (**B**) after fresco paint application; (**C**) after gypsum coat application; (**D**) PA image of (**C**); (**E**,**F**) NIR images of (**C**). The scale bar in (**A**) is valid for all the images.

Visible images of Sample 3 (Figure 4A–C) show yet another simulated scenario in which the underdrawing traits, made with sinopia and with a charcoal/sinopia mixture, are covered first with an Egyptian blue fresco paint (Figure 4B) and then with 80-micron thick gypsum layer (Figure 4C). Both PA and NIR images were processed in equal way by means of ImageJ, applying 1.0 contrast stretching with min value: 10.826. The PA image of the stratified sample, Figure 4D, reveals the mixed charcoal/sinopia trait whereas the sinopia lines are visible in NIR reflectograms (Figure 4E,F).

### 3.3. Imaging Charcoal and Sinopia Drawings under Limewash Coat: Samples 4 and 5

**Figure 5:**
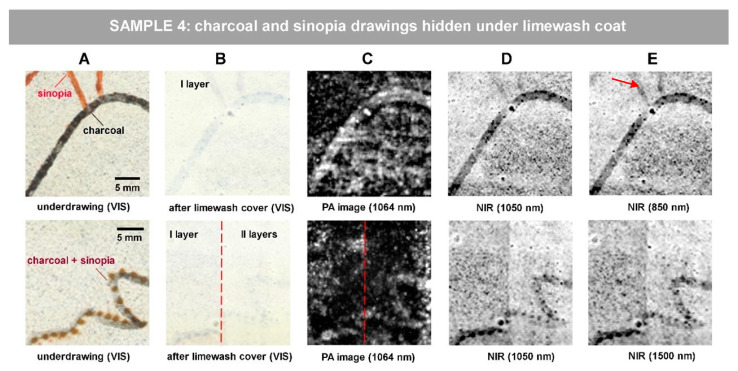
Revealing hidden sketch drawings under limewash coat in Sample 4: (**A**) visible image prior to limewash application; (**B**) visible image after limewash coat application; (**C**) PA imaging result; (**D**,**E**) NIR reflectance images. The scale bar in (**A**) is valid for all the images of the row.

The next step of our research was aimed at revealing the underdrawings under the limewash coat. Such simulation is represented in Sample 4, in which sinopia and charcoal traits are concealed by a 60-microns thick limewash coat (Figure 5A,B top raw) and mixed sinopia/charcoal under a 140 microns limewash (Figure 5A,B bottom raw). Both PA and NIR images were processed in equal way by the means of ImageJ, applying 1.0% contrast stretching and 0.9 gamma correction.

PA imaging reveals the charcoal underdrawing in both scenarios, also under two layers of gypsum, as displayed in Figure 5C. Sinopia underdrawing is detectable only by NIR reflectance imaging at lower wavelengths, at 1050 nm (Figure 5D) and best defined at 850 nm (Figure 5E). It is well known that depending on its concentration, sinopia becomes progressively transparent as a function of wavelength.

**Figure 6:**
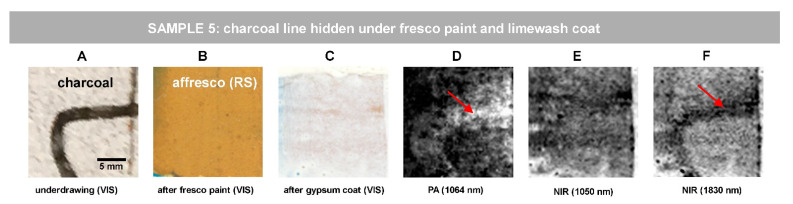
Revealing hidden sketch drawings under limewash coat in Sample 5: visible images of (**A**) initial charcoal sketch drawing; (**B**) after *fresco p*aint application; (**B**) after limewash coat application; (**C**) PA image; (**D**,**E**) NIR reflectance images. The scale bar in (**A**) is valid for all the images of the row. The red arrows in (**D**,**F**) indicate the blurred edges of the underdrawing, which may be the result of dispersion of charcoal particles when applying by brush the fresco layer.

For the abovementioned reason, we simulated stratified Sample 5, where two concealing layers (raw sienna fresco paint and 70-micron thick limewash coat) hide the charcoal traits (Figure 6A–C). Indeed, in such a sample, both PA (Figure 6D) and NIR (Figure 6E,F) imaging are capable of revealing the charcoal sketches. We present here the PA and NIR images processed in equal way by the means of ImageJ, applying 1.0% contrast stretching. The charcoal line edges appear slightly blurred which is probably attributable to the fresco execution method causing the dispersion of black pigment particles, as best revealed by PA image in Figure 6D and by NIR image at 1830 nm in Figure 6F. The best NIR result is observed at 1830 nm (Figure 6F), where limewash is known to show the highest transparency [20].

## 4. Discussion of the Results

We applied the novel epi-illumination PA imaging technique to ad-hoc designed wall painting mock-ups with concealed underdrawings and fresco layers: overpainted, covered by gypsum and limewash coats. The method proved powerful in revealing hidden sketch materials characterized by strong absorption properties at the working excitation wavelength of the instrument (1064 nm). Therefore, detection of graphite underdrawing is feasible with both PA and NIR techniques, whereas sinopia is detectable only by NIR at lower wavelengths (the best result at 850–950 nm), where this material shows stronger absorption properties. NIR imaging proved particularly useful in revealing both characteristic traditional fresco underdrawing materials (charcoal and sinopia) under thin hiding coats (<80 μm). This can be explained by the fact that in the near-infrared range, thin gypsum and lime layers scatter and absorb less light, which results in their increased transparency (when compared to the visible range). At the wall surface, however, the near-infrared radiation is reflected from the ground and absorbed by the underdrawing. In NIR reflectography, revelation of hidden drawings, located at the wall ground, is subject to both near-infrared transparency of hiding coats (due to low scattering and low absorbance) and to the differences in near-infrared reflection/absorption properties of the wall painting materials. While the performance of NIR imaging is subject to optical properties of the hiding and hidden features, the performance of PA imaging is based on the optical and acoustic properties of the materials. When increasing the coat thickness (up to 190 in gypsum and up to 140 in limewash coats), contrast enhancement both in PA and in NIR images becomes helpful for better revelation of charcoal traits. Revealing materials with a low absorption contrast at 1064 nm (e.g., sinopia) is expected to be addressed in future development of the reflection mode PA method at more excitation wavelengths.

We further note that the PA imaging complements the traditional NIR imaging technique in studying the peculiar features of charcoal traits, e.g., definition of the line, blurred edges, and *spolvero* dots. These details are helpful for the study of the execution technique, author attribution other art historical and conservation queries.

The effectiveness of both techniques is determined by the combination of the physical properties of constituent materials at the system operating wavelengths: optical properties for NIR imaging and both optical and ultrasonic properties for PA imaging. This difference can help understand divergences in the detectability of hidden features by the two techniques even at same thickness of hiding material and similar working wavelengths (e.g., between PA image at 1064 and NIR image at 1050 nm). We cannot exclude the fact that the result might be also influenced by the grain size and morphology of top layers [43], which is subject of further studies by authors.

This paper deals with the first proof-of-concept study and at the initial step, the use of water as an immersion medium, was preferred due to the simplicity of handling and due to its optimal bond performance for signal propagation. In the future, we expect further development of the technique with the implementation of air-coupled transducers, which have been already introduced in the transmission mode photoacoustic imaging set up [30]. Moreover, the same detection technology of PA signals has been recently employed for the investigation of restoration operations in a historical oil painting from the 19th century, demonstrating the high potential of air-coupled detectors in transmission geometries [44]. Similar implementations in reflection-mode may enable absolutely non-contact and non-invasive PA imaging of painted artifacts with a relative compromise as regards the detection sensitivity and the spatial resolution of the system.

## 5. Conclusions

Here, non-invasive PA imaging was tested for the first time tested as a tool for revealing hidden underdrawings in wall paintings with a complex stratigraphy. In this study, we exploited the prototype in its new epi-illumination configuration. The first experimental results presented here demonstrate the effectiveness of PA imaging in revealing hidden underdrawings in simulated retouched (overpainted) and concealed (by gypsum and limewash) wall paintings.

The non-invasive approach by the novel reflection-mode PA imaging technique proved capable of revealing the simulated hidden features in fresco and secco wall paintings, prepared following historical methodologies. The results were complemented and validated by the well-established NIR imaging technique. PA and NIR imaging proved complementary here in revealing hidden sketches (graphite, charcoal, sinopia, mix of charcoal and sinopia) under overpaint, gypsum and limewash coats, also in the presence of fresco paint layers. Under thicker concealing layers of gypsum (up to 190 μm), PA proved efficient in revealing charcoal traits (also in lower concentration, mixed with sinopia), showing a good contrast also under thicker concealing layers of gypsum. The current limitations of the novel reflectance photoacoustic imaging prototype, discussed above, are expected to be addressed in future development of the method. On the basis of the obtained results, we expect that this efficient non-invasive diagnostic tool will represent a breakthrough in Heritage Science for diagnostic applications to complex wall paintings, for their conservation and, if reasonable, for their safe uncovering.

## Figures and Tables

**Table 1:** Analyzed mock-ups.

Hidden Features	Hiding Layer		Sample Code
	Material	Measured Thickness	
Charcoal and graphite	tempera EB ^1^ paint	40–70 μm	1
Charcoal and sinopia	1 layer gypsum + glue	60–80 μm	2
	2 layers gypsum + glue	130–190 μm	
Charcoal and sinopia +fresco paint EB ^1^	1 layer gypsum + glue	60–80 μm	3
Charcoal and sinopia	1 layer limewash + milk	60–80 μm	4
	2 layers of limewash + milk	90–140 μm	
Charcoal + fresco paint RS ^2^	1 layer limewash + milk	60–80 μm	5

^1^ EB: Egyptian blue pigment; ^2^ RS: raw sienna pigment.

## Data Availability

The data that support the findings of this study are available from the corresponding author upon reasonable request.
